# Supplementary material for: ER-associated VAP27-1 and VAP27-3 proteins functionally link the lipid-binding ORP2A at the ER-chloroplast contact sites
Source: Nat Commun. 2024 Jul 17;15:6008. doi: 10.1038/s41467-024-50425-7 (PMC11255254; doi:10.1038/s41467-024-50425-7)
Supplement: Supplementary file 1 — Supplementary Information [file 41467_2024_50425_MOESM1_ESM.pdf]

Supplemental Figures

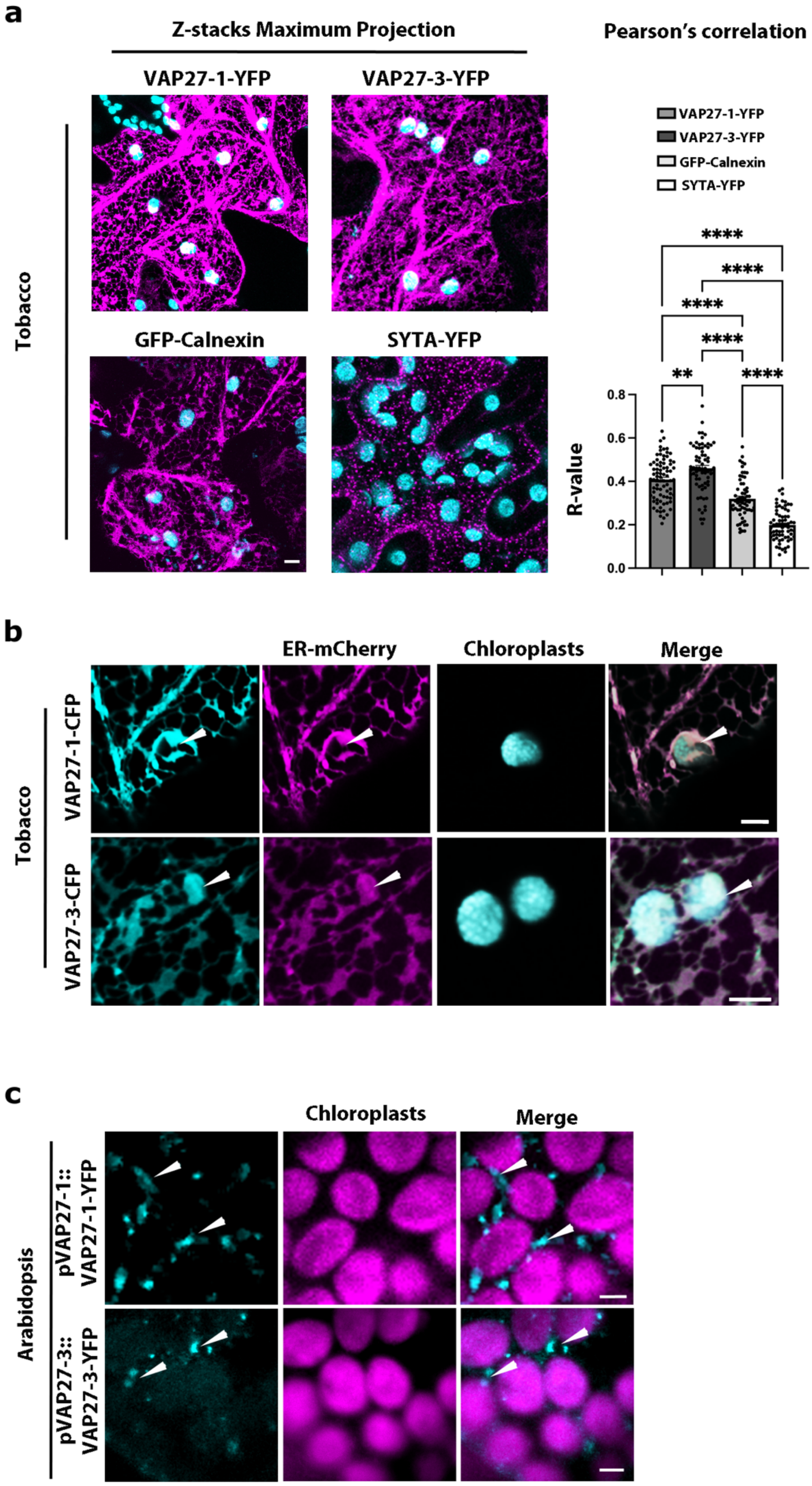

Supplementary Figure 1. The domain marked by the VAP27 proteins are in continuation with the bulk ER.

a. Z-stack maximum projections of tobacco epidermal cells transiently expressing VAP27-1-YFP, VAP27-3-YFP, GFP-Calnexin and SYTA-YFP show the different distribution of these proteins relative to multiple chloroplasts, which was evaluated by overlapping analysis and Pearson`s correlation coefficient analyses; VAP27-1-YFP (n=80), VAP27-3-YFP (n=70), GFP-Calnexin (n=62) and SYTA-YFP (n=68). Scale bar = 5 μm. **b**. Triple fluorochrome confocal live cell images of tobacco leaf epidermal cells co-expressing the ER lumenal marker ER-mCherry with either VAP27-1-CFP or VAP27-3-CFP show mCherry signal in the EMPC in close apposition to the chloroplasts (arrowheads). The latter are visible with the autofluorescence of the chlorophylls. Scale bars = 5 μm. **c**. Confocal images of Arabidopsis cotyledons stably expressing the cDNA of either VAP27 protein driven by the respective endogenous promoter (i.e., *pVAP27-1::VAP27-1-YFP*; *pVAP27-3::VAP27-3-YFP*). The VAP27 protein fusions accumulate in the ER in proximity of chloroplasts (arrowheads), which are visible through chlorophyll autofluorescence. Scale bars = 5 μm.

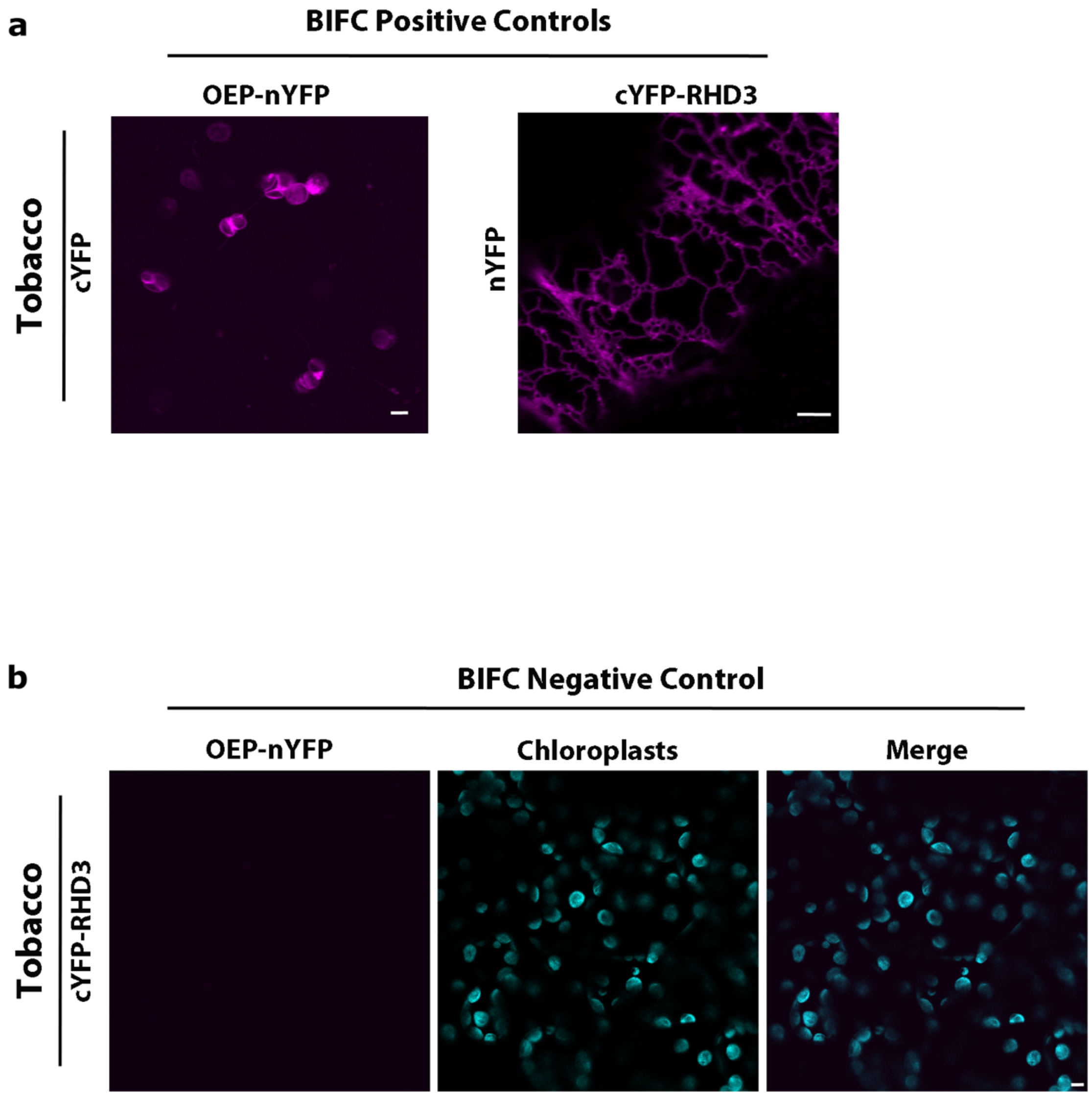

Supplementary Fig. 2. Positive and negative controls for BIFC analyses

a. Tobacco leaf epidermal cells transiently co-expressing OEP-nYFP and cYFP show signal from reconstituted fluorescence of OEP around the chloroplasts (left panel); while co-expression of cYFP-RHD3 and nYFP shows reconstituted fluorescence of RHD3 at ER level (right panel). Both represent positive controls for BIFC experiment shown in Figure 3. **b.** tobacco epidermal cells transiently co-expressing cYFP-RHD3 and OEP-nYFP, shows no fluorochrome reconstitution. This represents the negative control for BIFC experiment shown in Figure 3. Scale bar 5 μm

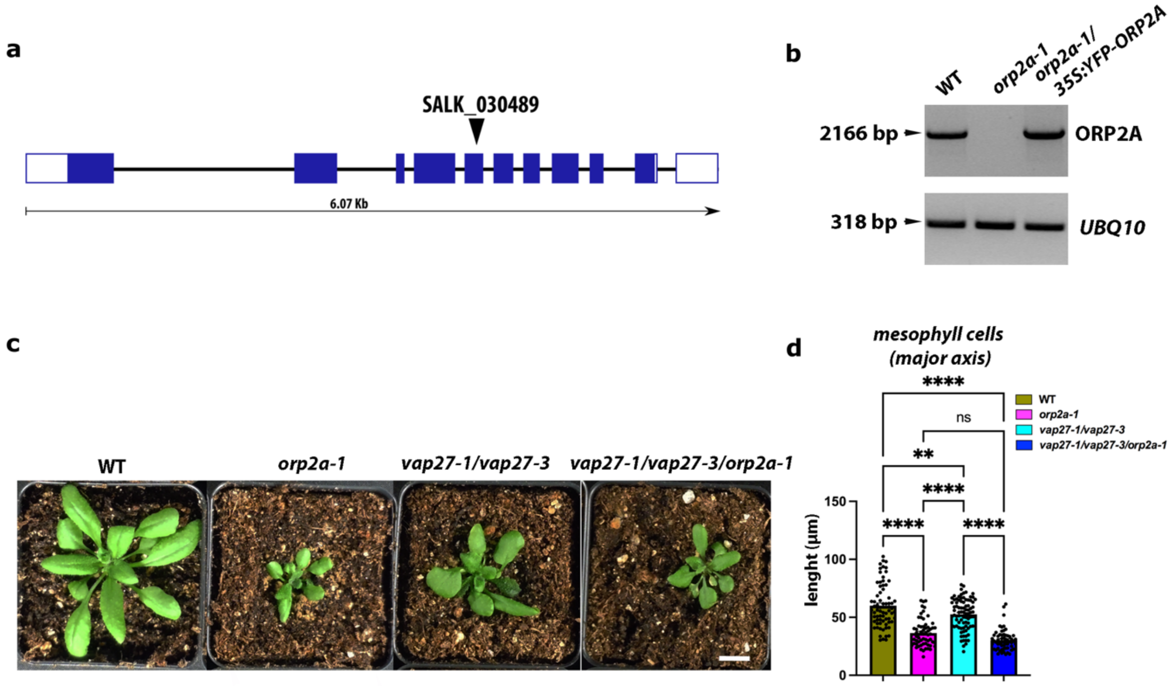

Supplementary Figure 3. Isolation of the *orp2a-1* allele and complementation analyses

a. Genomic DNA structure of the *ORP2A* locus (*At4g22540*) showing the position of the T-DNA insertion (SALK_030489). Blue rectangles: exons; line: introns, white rectangles: UTRs. **b**. RT-PCR of full-length *ORP2A* showing presence of transcripts in WT and complemented lines but not in the *orp2a-1* allele. *UBQ10*: PCR control, number of cycles n=35. **c**. Picture of the rosettes of 4 weeks-old plants of WT, *orp2a-1*, *vap27-1/vap27-3* and *vap27-1/vap27-3/orp2a-1,* scale bar=1 cm*)* **d.** Graph representing the measurements of mesophyll cells major axis (cell diameter) for WT (n=71), *orp2a-1*(n=61), *vap27-1/vap27-3* (n=85) and *vap27-1/vap27-3/orp2a-1* (n=59). $^{*}P < 0.05$, $^{**}P < 0.01$, $^{***}P < 0.001$, $^{****}P < 0.0001$ (p-value calculated with one-way Anova with Tukey's post test)

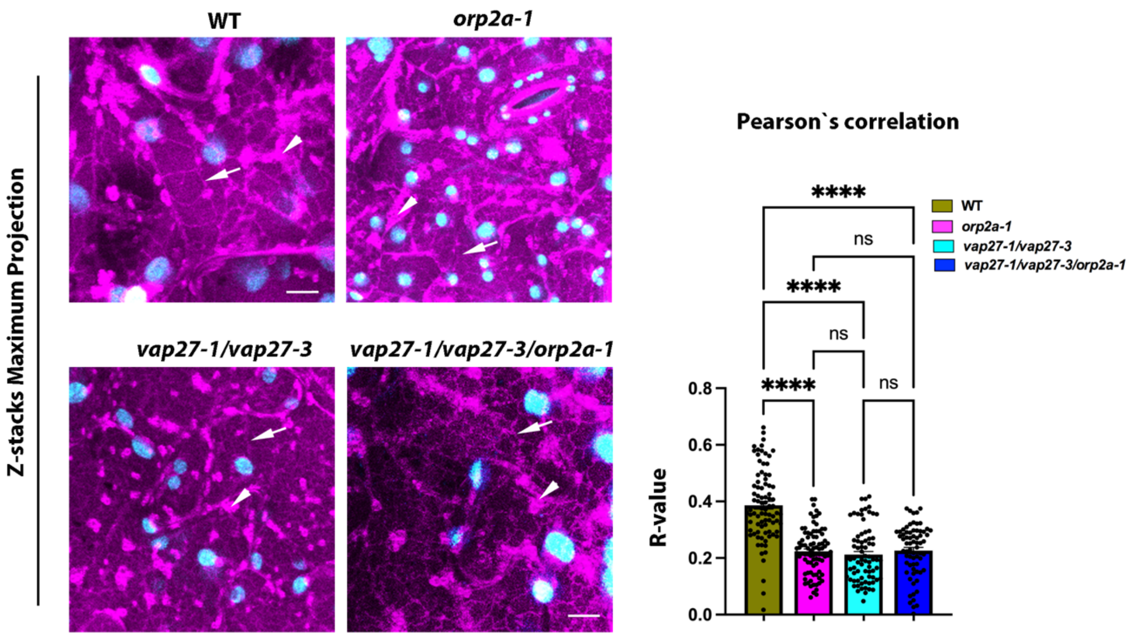

Supplementary Figure 4. ER labelling by DiOC6 in WT, *orp2a-1, vap27-1/vap27-3* and *vap27-1/vap27-3/orp2a-1*

Z-stacks maximum projections of WT, *orp2a-1, vap27-1/vap27-3* and *vap27-1/vap27-3/orp2a-1* leaf epidermal cells labelled at ER level with DiOC6 in magenta. ER signal overlapping with chloroplasts (chlorophyll autofluorescence in cyan) is quantified and compared in all the lines by Pearson`s correlation coefficient. Arrows indicate ER tubules, while arrowheads indicate ER cisternae. WT (n=85), *orp2a-1* (n=86), *vap27-1/vap27-3* (n=75), and *vap27-1/vap27-3/orp2a-1* (n=69), *$P < 0.05$, **$P < 0.01$, ***$P < 0.001$, ****$P < 0.0001$ (p-value calculated with one-way Anova with Tukey's post test)

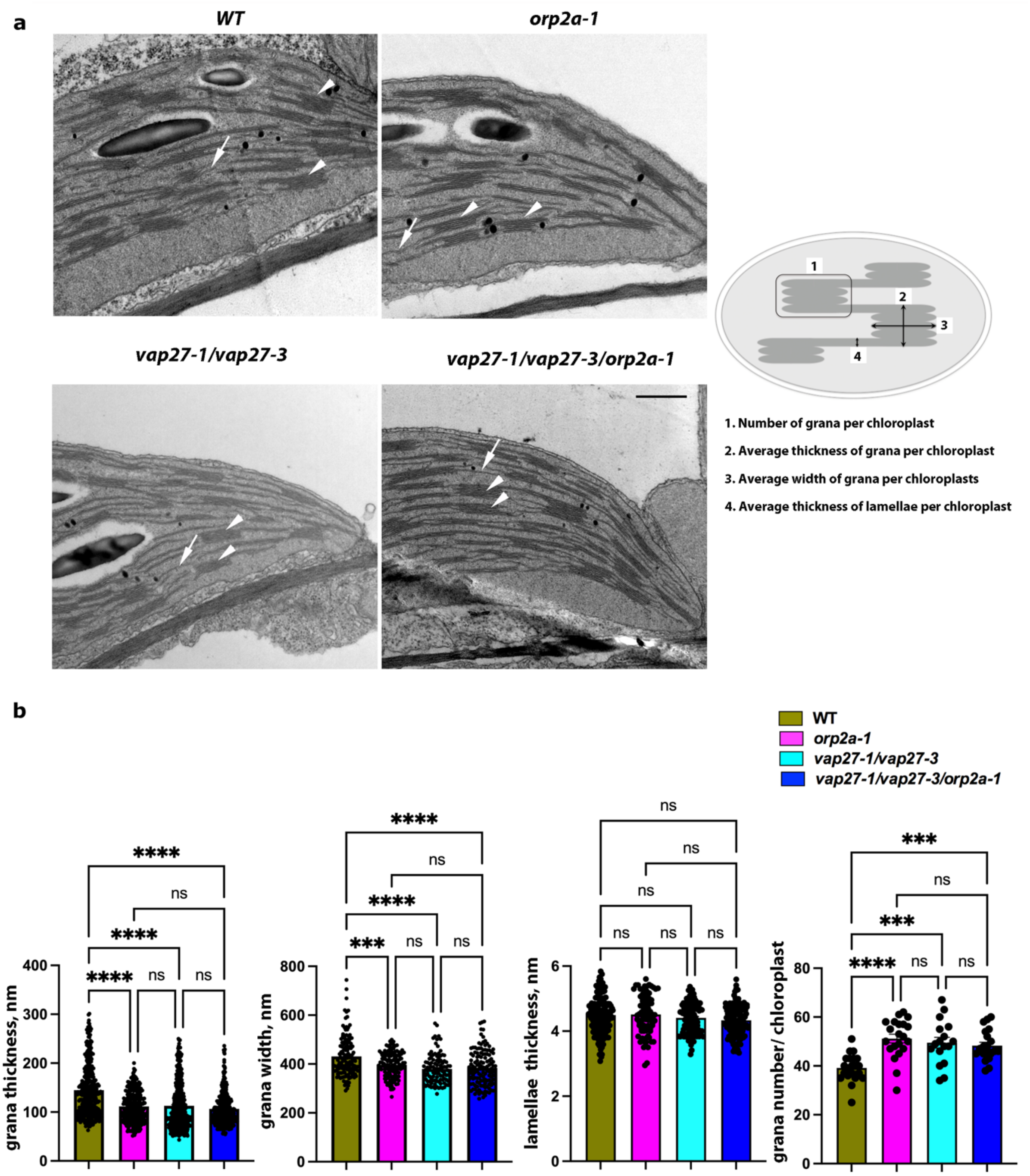

Supplementary Figure 5. Ultrastructural analysis of the chloroplasts of WT, *orp2a-1, vap27-1/vap27-3* and *vap27-1/vap27-3/orp2a-1*

a. Transmission electron micrographs of chloroplasts of WT, *orp2a-1, vap27-1/vap27-3* and *vap27-1/vap27-3/orp2a-1* showing grana (arrowheads) and lamellae (arrows). The diagram represents how the images were analyzed for measuring grana thickness, length and number, and lamellae thickness in all the lines. Scale bar = 500 nm. **b.** Quantification of grana thickness, length and number, and lamellae thickness in all the lines. Error bars: S.E.M. $^{*}P < 0.05$, $^{**}P < 0.01$, $^{***}P < 0.001$, $^{****}P < 0.0001$(p-value calculated with one-way Anova with Tukey's post test)

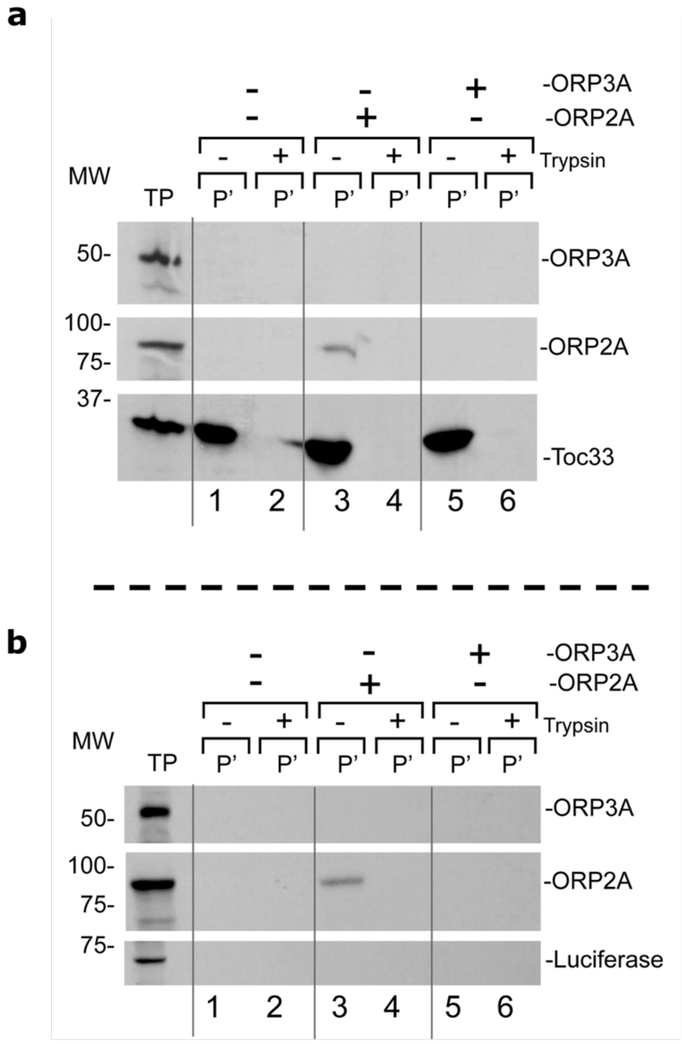

Supplementary Figure 6. The association with the OEM is specific to ORP2A

a. Upper panel, SDS-PAGE and fluorography analysis of *in vitro* association assays using pea chloroplasts shows that [^{3}H]ORP2A was recovered only in the absence of trypsin treatment, while [^{3}H]ORP3A was not recovered in the absence of trypsin treatment. [^{3}H]Toc33, an OEM control protein, associates with the OEM and is trypsin sensitive, in a similar fashion as ORP2A which specifically associates with the OEM while ORP3A does not associate with the OEM. **b.** Lower panel, SDS-PAGE and fluorography analysis show that [^{3}H]ORP2A specifically associates with the OEM whereas both [^{3}H]ORP3A and [^{3}H]Luciferase, a nonspecific control protein, do not associate with the OEM. TP= 10% of Translation Product added to association assay.

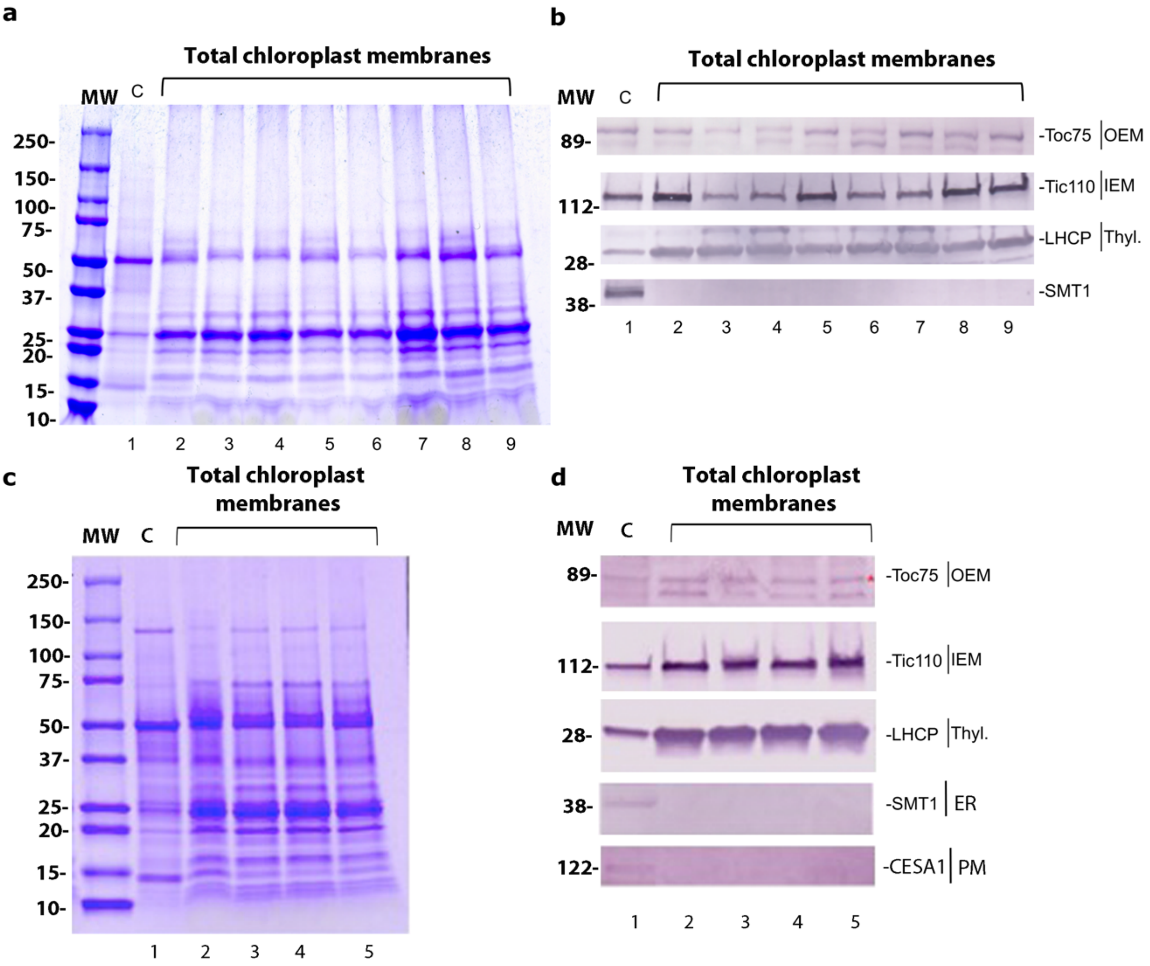

Supplementary Figure 7. Full-size Western blots of the extracts of purified chloroplasts

a and **b** represent full-size gel and Western blots of the extracts of the purified chloroplasts presented in Figure 7b. Coomassie SDS-PAGE (**c**) and Western blot analyses (**d**) represent full size gel and Western blot of the chloroplast extracts presented in Figure 7c, relative to CESA1 (PM marker) detection. The extracts were loaded on the gel as follows:1) Col-0 total leaf control; and total chloroplast membranes (5 mg chlorophyll/lane) from the following lines: 2) WT, 3) *orp2a-1*; 4) *vap27-1/vap27-3*, and 5) *vap27-1/vap27-3/orp2a-1* were resolved by 4-20% SDS-PAGE (Bio-Rad™ precast gel) and either Coomassie Stained (**c**) or subjected to Western blot Analysis (**d**). Western blots in (**d**) were probed with either anti-Toc75 (N-terminal region), anti-Toc33, anti-Tic110, anti-LHCB1/3 (N-terminal region), anti-SMT1, and anti-CESA1.

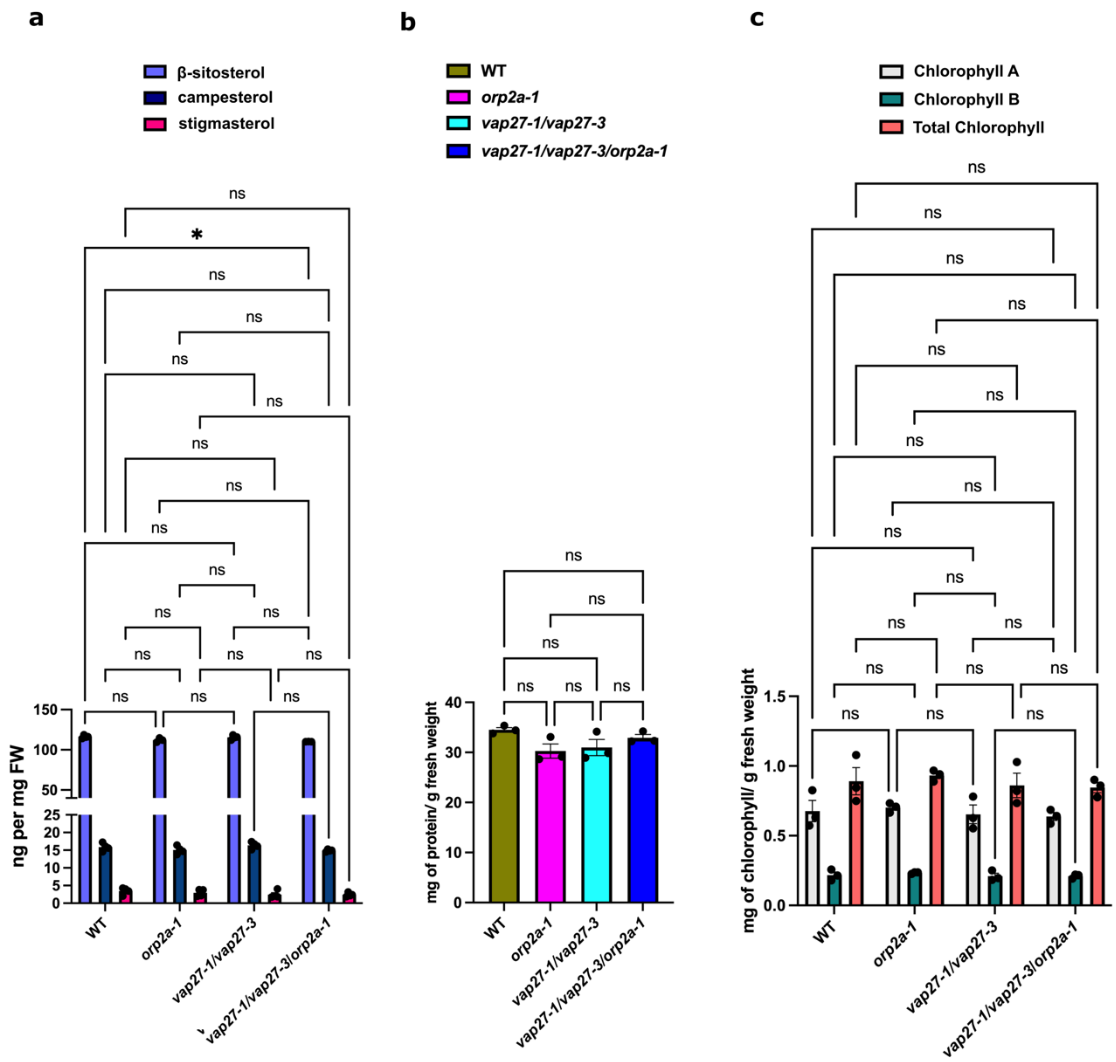
a
β-sitosterol
campesterol
stigmasterol
ng per mg FW
WT
orp2a-1
vap27-1/vap27-3
vap27-1/vap27-3/orp2a-1
b
WT
orp2a-1
vap27-1/vap27-3
vap27-1/vap27-3/orp2a-1
mg of protein/ g fresh weight
c
Chlorophyll A
Chlorophyll B
Total Chlorophyll
mg of chlorophyll/ g fresh weight

Supplementary Figure 8. Total phytosterols leaf extract levels are not affected in the mutants compared to the WT

a. Quantification of total of β-sitosterol, campesterol and stigmasterol levels in leaves of WT, *orp2a-1*, *vap27-1/vap27-3*, *vap27-1/vap27-3/orp2a-1*. Only slight differences were identified in the quantification of β-sitosterol between WT and the triple mutant (*$P < 0.05$). The data were normalized to the total protein content (**b**; mg of protein/g fresh weight). **c**. Quantification of chlorophylls (mg chlorophylls/g fresh weight) in the analyzed genotypes. No significant differences were detected in the protein levels (**b)** and chlorophylls levels (**c**) across the genotypes.

Supplementary Table1: Reagents and Primers

REAGENT or RESOURCE	SOURCE	IDENTIFIER
**Antibodies**		
Anti_CESA1	PhytoAB	PHY0798S
anti-His	Santa Cruz biotechnology	sc-804
anti-GST	ThermoFisher Scientific	A-5800
Lhcb1	Agrisera	AS09522
SMT1	Agrisera	AS07266
secondary antibody (goat anti-rabbit IgG-alkaline phosphatase conjugate	ThermoFisher	G-21079
**Bacterial and Virus Strains**		
Agrobacterium tumefaciens GV3101	n/a	n/a
E. coli TOP10	Invitrogen™	C404006
E.coli BL21 DE3	Invitrogen™	C600003
**Chemicals, Peptides, and Recombinant Proteins**		
5α-cholestane	Millipore Sigma	47124
3β-Hydroxy-5α-cholestane	Millipore Sigma	47129
β-sitosterol	Millipore Sigma	43623
Stigmasterol	Millipore Sigma	47132
Ergosterol	Millipore Sigma	47130
Cholesterol	Echelon Biosciences	L-6012
phosphatidylglycerol (PG)	Echelon Biosciences	L-5116
Phosphatidylinositol (PI)	Echelon Biosciences	P-0016
Phosphatidylinositol 3-phosphate PI(3)P	Echelon Biosciences	P-3016
MGDG	Millipore Sigma	840526P
DGDG	Millipore Sigma	840527P
Methanol, HPLC grade	Millipore Sigma	439193-4L
Chloroform, HPLC grade	Millipore Sigma	650498-1L
Hexane, HPLC grade	Millipore Sigma	1043352500
Butylated hydroxytoluene (BHT)	Millipore Sigma	1082708-500MG
BSTFA (with 1% TMCS)	Millipore Sigma Catalog	B-023-10x1mL
Latrunculin B	Calbiochem	428020
IMIDAZOLE, REAGENTPLUS, 99%	Merck-sigma	I202-500G
BOVINE SERUM ALBUMIN, HEAT SHOCK FRACT&	Merck-sigma	A7030-10G
B-SITOSTEROL SYNTHETIC APPROX. 95%	Merck-sigma	S1270-10MG

CAMPESTEROL	Merck-sigma	PHL89514-10MG
cOmplete(TM), Mini, EDTA-free Protease I	Merck-sigma	11836170001
STIGMASTEROL	Merck-sigma	S2424-1G
OmniPur® IPTG [Isopropyl-ß-D-Thiogalactopyranoside]	Merck-sigma	5800-1GM
1-StepTM NBT (nitro-blue tetrazolium chloride) /BCIP (5-bromo-4-chloro-3'-indolyphosphate p-toluidine) solution	ThermoScientific	34042
**Critical Commercial Assays**		
**Experimental Models: Organisms/Strains**		
Sail_355_E10_LP	Tair	
WiscDsLoxHs096_06H	Tair	
Salk_030489	Tair	
Arabidopsis thaliana Col3		
Arabidopsis thaliana Col0		
**Oligonucleotides**		
ATGAGTAACATCGATCTGATTGGGATG	Invitrogen	F-At3g60600-5’
TGTCCTCTTCATAATGTATCCCAA	Invitrogen	R-At3g60600-3’ full length for c terminal fusion
ATGAGTAACGAGCTTCTCACCATCGAT	Invitrogen	F-At2g45140-5’
TCATGTCCTCTTCATAATGTATCCCAGG	Invitrogen	R-At2g45140-3’ full length for c terminal fusion
GATATACATATGAGTAACATCGATCTGATTGGGATGAG	Invitrogen	F-At3g60600-5’
GTGGTGCTCGAGTTAACCACTTTGGCTCTTTTTGCTTTCACGC	Invitrogen	R-At3g60600-3’
GCAGATCTCTTGTTCTCTCTATTTGGGT	Invitrogen	F- At2g45140 specific primer
CCCTGTCTGCGCTAAATCTTGGAG	Invitrogen	R-At2g45140 specific primer
GATATACAT ATGAGTAACATCGATCTGATTGGGATGAG	Invitrogen	F-At3g60600 for NdeI cut and pET-16b vector cloning
GTGGTGCTCGAGTTAACCACTTTGGCTCTTTTTGCTTTCACGC	Invitrogen	R-At3g60600 for XhoI cut and pET-16b vector cloning
AGTTGTCTACGAGGCTTACCGAATCTTGCAACT	Invitrogen	F-native promoter Vap27-1
CCATTACATGGTAATTAATCAGTTCACCTCC	Invitrogen	F-native promoter VAP27-3
TCAATTCTCTCTACCGTGATCAAGATGCA	Invitrogen	F-UBI10
GGTGTCAGAACTCTCCACCTCAGAGTA	Invitrogen	R-UBI10
TAC CCC TAA TAA CGC ACC ATG	Invitrogen	Sail_355_E10_LP
TTA TCC TGA GCA TCA ACC CTG	Invitrogen	Sail_355_E10_RP
AAACAGAAGCCAATGCAAAAG	Invitrogen	WiscDsLoxHs096_06H_LP
GTTCTCCCGAGGTCTACTTGC	Invitrogen	WiscDsLoxHs096_06H_RP
TGATCCATGTAGATTTCCCGGACATGAAG	Invitrogen	BP- wisc ds lox HS
CATCTTTCACGTGGATGTGTG	Invitrogen	salk ORP2A Salk_030489 LP
CTCCATCATCGAACTCCAGAG	Invitrogen	salk ORP2A Salk_030489 RP

ATTTTGCCGATTTCGGAAC	Invitrogen	BP primer for Salk t-DNA insertion line
GCTTCCTATTATATCTTCCCAAATTACCAATACA	Invitrogen	BP primer for Sail t-DNA insertion line
CACC ATG GGG TCT CTC GTT CGT GAA TGG GTT	Invitrogen	F-AtToc33
TTA AAG TGG CTT TCC ACT TGT CTT GAT ATC ATT TCT	Invitrogen	R-AtToc33
CACC ATG GAA GAC GCC AAA AAC ATA AAG AAA	Invitrogen	F-Luciferase
TTA CAC GGC GAT CTT TCC GCC CTT CTT GGC	Invitrogen	R-Luciferase
CACC ATG GCT TCC TCT ATG CTC TCC TCT GCC	Invitrogen	F-AtSSU1B
TTA AGC ATC AGT GAA GCT TGG GGG CTT GTA	Invitrogen	R-AtSSU1B
CACC ATG GCT TCT AAC GAT CCA AAA AAC GGC	Invitrogen	F-ORP3A
TTA AGC AGA GAG ATCT TGG AAT	Invitrogen	R-ORP3A
CACC ATG CGG GTT AAA GAG TTA CAT CCA	Invitrogen	F-ORP2A
CTA AGC GGC GTC CGC TAG CTC TTC TGT GAA	Invitrogen	R-ORP2A
CACC ATG AGT AAC ATC GAT CTG ATT GGG	Invitrogen	F- AtVAP27-1(ΔTMD)His
TTA GTG GTG GTG GTG GTG GTG TTG GCT CTT TTT GCT TTC ACG	Invitrogen	R- AtVAP271(ΔTMD)His
CACC ATG AGT AAC GAC CTT CTC ACC ATC GAT	Invitrogen	F-AtVAP27-3(ΔTMD)His
TCA GTG GTG GTG GTG GTG GTG CTT ACT TCT CTT GCT TTC GCG CCT	Invitrogen	R-AtVAP27-3(ΔTMD)His
**Recombinant DNA**		
VAP27-1-YFP		
Vap27-3-YFP		
CNX-GFP		
VAP27-1-CFP		
VAP27-3-CFP		
cYFP-VAP27-1		
cYFP-VAP27-3		
OEP-nYFP		
GST-VAP27-1		
GST-VAP27-3		
HIS-ORP2A		
pVAP27-1::VAP27-1YFP		
pVAP27-3::VAP27-3YFP		
YFP-ORP2A		
SYTA-YFP		
**Software and Algorithms**		
ImageJ	NIH	https://imagej.nih.gov/ij/
Graphpad Prism	Graphpad	https://graphpad.com

NIS-Elements AR	Nikon	https://www.nikoninstruments.com/Products/Software/NIS-Elements-Advanced-Research
AdobePhotoshop	Adobe	https://www.adobe.com/products/photoshop.html
